# *Ptpn20* deletion in H-Tx rats enhances phosphorylation of the NKCC1 cotransporter in the choroid plexus: an evidence of genetic risk for hydrocephalus in an experimental study

**DOI:** 10.1186/s12987-022-00341-z

**Published:** 2022-06-03

**Authors:** Hanbing Xu, Masakazu Miyajima, Madoka Nakajima, Ikuko Ogino, Kaito Kawamura, Chihiro Akiba, Chihiro Kamohara, Koichiro Sakamoto, Kostadin Karagiozov, Eri Nakamura, Nobuhiro Tada, Hajime Arai, Akihide Kondo

**Affiliations:** 1 Department of Neurosurgery, Juntendo University Graduate School of Medicine, Hongo Bunkyo-ku, Tokyo, 113-8421 Japan; 2 Department of Neurosurgery, Juntendo Tokyo Koto Geriatric Medical Center, 3-3-20 Shinsuna, Koto-ku, Tokyo, 136-0075 Japan; 3 Department of Genetic Analysis Model Laboratory, Juntendo University Graduate School of Medicine, Hongo Bunkyo-ku, Tokyo, 113-8421 Japan

**Keywords:** Cerebrospinal fluid, Choroid plexus, H-Tx rats, Hydrocephalus, NKCC1 cotransporter, Ventriculomegaly

## Abstract

**Background:**

Congenital hydrocephalus occurs with some inheritable characteristics, but the mechanisms of its development remain poorly understood. Animal models provide the opportunity to identify potential genetic causes in this condition. The Hydrocephalus-Texas (H-Tx) rat strain is one of the most studied animal models for investigating the causative genetic alterations and analyzing downstream pathogenetic mechanisms of congenital hydrocephalus.

**Methods:**

Comparative genomic hybridization (CGH) array on non-hydrocephalic and hydrocephalic H-Tx rats was used to identify causative genes of hydrocephalus. Targeted gene knockout mice were generated by CRISPR/Cas9 to study the role of this gene in hydrocephalus.

**Results:**

CGH array revealed a copy number loss in chromosome 16p16 region in hydrocephalic H-Tx rats at 18 days gestation, encompassing the protein tyrosine phosphatase non-receptor type 20 (*Ptpn20*), a non-receptor tyrosine phosphatase, without change in most non-hydrocephalic H-Tx rats. *Ptpn20*-knockout (*Ptpn20*^*−/−*^) mice were generated and found to develop ventriculomegaly at 8 weeks. Furthermore, high expression of phosphorylated Na-K-Cl cotransporter 1 (pNKCC1) was identified in the choroid plexus (CP) epithelium of mice lacking *Ptpn20* from 8 weeks until 72 weeks.

**Conclusions:**

This study determined the chromosomal location of the hydrocephalus-associated *Ptpn20* gene in hydrocephalic H-Tx rats. The high level of pNKCC1 mediated by *Ptpn20* deletion in CP epithelium may cause overproduction of cerebrospinal fluid and contribute to the formation of hydrocephalus in *Ptpn20*^*−/−*^ mice. *Ptpn20* may be a potential therapeutic target in the treatment of hydrocephalus.

**Supplementary Information:**

The online version contains supplementary material available at 10.1186/s12987-022-00341-z.

## Introduction

Hydrocephalus represents excess accumulation of cerebrospinal fluid (CSF) in the cerebral ventricles and subarachnoid space. In humans, the prevalence of congenital hydrocephalus is high and markedly geographically variable. In sub-Saharan Africa, there are approximately 750 new cases per 100,000 live births, whereas in Europe, there are only 110 cases of infantile hydrocephalus per 100,000 live births[1]. Congenital hydrocephalus is the most common neurological disorder requiring surgery in children [2]. Despite the high prevalence of congenital hydrocephalus, knowledge about the pathophysiological mechanisms leading to this disorder remains extremely limited. A large body of evidence suggests that congenital hydrocephalus is an inheritable disorder and characterization at the molecular level should greatly increase the understanding of the disease and lead to new therapies. However, relatively few genes in humans, including *L1CAM*, *MPDZ*, and *CCDC88C* have been linked with hydrocephalus to date [3–5]. Less than 5% of mutations in these genes account for primary congenital hydrocephalus cases [6]. As animal models of inherited disease provide opportunities to identify potential genetic causes of disease in humans, a variety of genes causing hydrocephalus in rodents have been identified and characterized over the past decades in transgenic animal models, including the Hydrocephalic-Texas (H-Tx) rat, the Wistar-Lewis rats, the hydrocephalus-1 mouse, the hydrocephalus with hop gait mouse, and the TGF-beta1 mouse [7–13].

Among these, the H-Tx rat strain, first described in 1981, with congenital hydrocephalus resulting from a spontaneous mutation is widely used as a model of the pathogenesis of the disease [14]. Hydrocephalus in H-Tx rats develops in early gestation via a complex mode of inheritance and with a prevalence of 30–50% [15]. Ventricular dilatation starts to occur from days 17 to 18 of gestation and is associated with aqueductal stenosis [16, 17]. Pups with severe hydrocephalus are detected at birth by a domed head and die at 4–6 weeks of age. Breeding data from crosses between H-Tx rats and Sprague-Dawley (SD) rats have indicated that hydrocephalus in H-Tx rats is a single autosomal recessive gene disease [18], but a series of studies by Jones et al. indicated that the phenotypic expression of congenital hydrocephalus in them is controlled by a combination of genetic and epigenetic factors [15, 19–23].

The genetic abnormality causing congenital hydrocephalus in H-Tx rats remains only partially understood. In this study, we performed copy number analysis to investigate candidate genes potentially responsible for hydrocephalus in H-Tx rats. Under this approach, we identified a copy number loss in the *Ptpn20* gene in hydrocephalic H-Tx rats, generated *Ptpn20-*knockout (*Ptpn20*^*−/−*^) mice and then tested them for genotypic and phenotypic differences from the wild-type (WT) mice. Our study found significant differences in the expression of phosphorylated Na-K-Cl cotransporter 1 (pNKCC1) in the choroid plexus (CP) of *Ptpn20*^*−/−*^ mice compared to the WT. NKCC1 is encoded by Slc12a2 and belongs to the SLC12 family of cation-chloride cotransporters, which show a wide distribution in different tissues and cell types. In most secretory epithelial cells NKCC1 localizes at the basolateral membrane and mediates chloride transport as an important mechanism of cell volume regulation [24, 25]. In contrast, in the central nervous system (CNS), cotransporters are atypically located on the apical membrane of the CP [26, 27]. Because of this, the function of NKCC1 in the CP epithelium is unclear, and whether NKCC1 functions primarily as a secretory, permissive, or absorptive (clearance) fluid system there has been highly controversial [28–30]. Based on our findings, we suggest a hypothesis about the function of NKCC1 and propose a pathophysiological mechanism that may be related to the development of hydrocephalus and together with a possible therapeutic target.

## Materials and methods

### Animals

All experimental animals were group-housed with 2–5 animals per cage and maintained in a temperature- and humidity-controlled facility (23 ± 1 °C, 55 ± 5% humidity) on a 12-h light/dark cycle at our animal care facility in the Center for Experimental Medicine of Juntendo University, Japan. All procedures involving animals were approved by the Ethics Review Committee for Animal Experimentation of the Juntendo University School of Medicine (rats: approval no. 288; mice: approval no. 1337) and followed the Principles of Laboratory Animal Care as outlined by the National Institutes of Health.

Study animals used in genetic identification included hydrocephalic H-Tx (H-Tx (+)) rats and non-hydrocephalic H-Tx (H-Tx (−)) rats at 18 days gestation (E18), 1 day postnatal (P1) and 1 week postnatal (P7), and Wistar and SD rats at P1. Among these, E18 H-Tx (+) and H-Tx (−) rats were differentiated from the size of the ventricles on tissue sections, and the P1 H-Tx (+) and H-Tx (−) rats—by the characteristic “domed head” appearance on gross examination (Additional file 1: Fig. S1A, B). H-Tx (−). Wistar and SD rats served as controls.

C57BL/6J mice were used to analyze *Ptpn20* expression and generate *Ptpn20*^*−/−*^ mice by CRISPR/Cas9 technology (described below), evaluating them at 4, 8, 24, 48, and 72 weeks of age.

All animals were decapitated for dissection of the brain after induction of deep anesthesia using intraperitoneal pentobarbital (50 mg/kg body weight). Each brain sample was immediately stored in RNA*later*® solution (AM7021; Thermo Fisher Scientific, Waltham, MA, USA) for DNA, RNA and protein extractions.

### Identification of genetic risk in H-Tx rats

#### Comparative genomic hybridization array

DNA was extracted from the brain of H-Tx (+) rats and H-Tx (−) rats at E18, and genome-wide DNA copy number analysis was performed using a SurePrint G3 Rat Comparative genomic hybridization array (CGH) Microarray kit 1 × 1 M SG13464375 (ID-027065; Agilent Technologies, Santa Clara, CA, USA) according to the protocol from the manufacturer. Data were extracted using Feature Extraction version 10.1.1.1 software (Agilent Technologies), and analyses were performed using Agilent Genomic Workbench Standard Edition version 5.0.14 software (Statistical algorithm ADM-2, threshold of 6.0, fuzzy zero correction). Non-redundant copy number abnormalities were called as a minimum of three consecutive probes and log2 ratio > 0.3 for gains and < − 0.3 for losses were considered significant.

#### Real-time PCR of copy number

Copy number quantification was performed by TaqMan^®^ quantitative polymerase chain reaction on the brain tissues of E18 H-Tx (+) and H-Tx (−) rats using an ABI7500 real-time PCR system (Applied Biosystems, Thermo Fisher Scientific). TaqMan Copy Number assays (*Ptpn20* exon 6–7, Custom ID: CC70L9K, CCLJ15L) were run simultaneously with TaqMan Copy Number Reference assays [*Rnase11* (Rn02349567_s1), *Ftmt* (Rn01492073_s1) and *Hus1* (Rn02115575_s1)] (Applied Biosystems) according to the instructions from the manufacturers. Copy number variations were analyzed using ABI7500 software (Applied Biosystems) and Copy Caller version 2.0 software (Applied Biosystems).

#### Real-time PCR of *Ptpn20* mRNA

Total RNA (500 ng) was converted into single-stranded cDNA using SuperScript™ IV VILO™ (SSIV VILO) Master Mix (Invitrogen, Thermo Fisher Scientific). The ABI 7500 Real-time PCR System (Applied Biosystems) and TaqMan® Gene Expression Assays (Applied Biosystems) were used according to the instructions from the manufacturer to quantify gene expression. Assay IDs are listed in Additional file 5: Table S2.

Expressions of target genes were standardized to the expression of *Actb*. The presence of a single PCR amplicon was confirmed by melting curve analysis. Expressions of each gene in each sample were analyzed in triplicate.

#### Protein expression of *Ptpn20*

Proteins were extracted from CP (Additional file 6: Table S3) and lysed in 50 µl of lysis buffer (N-PER; Thermo Fischer Scientific) containing protease inhibitor cocktail (cOmplete ULTRA Mini EDTA-free EASYpack; Roche, Basel, Switzerland). Lysates were clarified by centrifugation at 20,000×*g* at 4 °C for 10 min, and protein concentrations of the resultant supernatants were determined using BCA Protein Assay Kits (Thermo Fischer Scientific). After 10–15 µg of proteins was heated at 70 °C for 10 min in NuPAGE® LDS Sample Buffer (NP0008; Invitrogen) and NuPAGE® Sample Reducing Agent (NP0009; Invitrogen), samples were electrophoresed on 4–12% NuPAGE® Bis-Tris Mini Gel by NuPAGE® MOPS SDS Running Buffer (20×) (NP0001; Invitrogen) Running Buffer (20×), and then transferred to a polyvinylidene fluoride membrane. Primary antibodies are listed in Additional file 6: Table S3. Signals were detected by chemiluminescence using a WesternBreeze kit (WB7103; Invitrogen). Immunoreactive bands were detected using ImageLab version 4.1 software (Bio-Rad Laboratories, Hercules, CA, USA).

#### Immunofluorescence of the CP

Animal brains were removed and postfixed with 4% paraformaldehyde in 0.01-M phosphate buffer (pH 7.2). Paraffin-embedded sections (4 μm) and cryosections (30 μm) were blocked with 5× SEA BLOCK™ blocking buffer (37527; Thermo Fisher Scientific) and 1% donkey serum in Phosphate buffered saline (PBS; AJ9P003; TaKaRa, Shiga, Japan) for 30 min, incubated in primary antibody overnight at 4 °C, and secondary antibodies for 60 min at room temperature. Vibratome Sect. (300 μm) were blocked using the same blocking buffer with 0.05% TritonX and 1% donkey serum in PBS for 30 min, then incubated in primary antibody for 2 days at 4 °C, and secondary antibodies for 2 h at room temperature. Primary and secondary antibodies are listed in Additional file 7: Table S4. Nuclei in all sections were counterstained with ProLong Gold and SlowFade Gold Antifade Reagent with DAPI (4′,6-diamidino-2-phenylindole; P36935; Molecular Probes®, Invitrogen). Images were acquired with a confocal scanning microscope (TCS-SP5; Leica Microsystems, Wetzlar, Germany). Leica Application Suite Advanced Fluorescence Lite (Leica Microsystems) was used for image acquisition and processing.

### Expression and role of *Ptpn20* in mice

#### Immunohistochemistry

Brain and six other tissues (testis, kidney, heart, pancreas, liver and spleen) of 4-week-old C57BL/6J mice were fixed in 4% paraformaldehyde fixative (33111; Muto Pure Chemicals Co., Tokyo, Japan) for at least 1 week, embedded in paraffin, and cut into 6-µm sections. Endogenous peroxidase was blocked by incubation of brain sections with 0.3% hydrogen peroxide for 30 min. Sections were blocked with 5× SEA BLOCK Blocking Buffer and 1% donkey serum in PBS at 25 °C for 30 min. Sections were incubated with rabbit PTPN20A antibody (CSB-PA199334, 1:50 dilution; CUSABIO, Houston, TX, USA) overnight at 4 °C. The following day, sections were incubated with EnVision™ System Labeled Polymer (DAKO, Glostrup, Denmark) as the secondary antibody for 30 min at room temperature. Finally, sections were stained with 3,3′-diaminobenzidine and counterstained with Mayer’s hematoxylin, dehydrated, cleared, and mounted. Sections were viewed under an E800 microscope (Nikon, Tokyo, Japan) and images were captured with an AxioCam 506 color digital camera using AxioVison Rel version 4.7.2.0 image-processing software (Carl Zeiss Microimaging GmbH, Jena, Germany).

#### Generation of *Ptpn20****-***knockout mice

Knockout mouse lines were generated using a CRISPR/Cas9 system in C57BL/6J mice with the single guide RNA (sgRNA) sequence (exon 4; CCTGAATCTCCGCAACTCTTTGC, underlined is the protospacer adjacent motif sequence from GRCm38.p6).

Both sgRNA and Cas9 protein were injected into the cytoplasm of fertilized one-cell eggs using continuous pneumatic pressure. Eggs were cultured in modified Whitten’s medium for approximately 24 h and developed to 2-cell stage embryos. Two-cell stage embryos were transferred into each oviduct of pseudopregnant Institute of Cancer Research (ICR) recipient females that had been mated to vasectomized ICR male mice. Embryo transfer to pseudopregnant females was performed on the day the vaginal plug was detected. After birth, genomic DNA was extracted from tail tips of mutant F0 mice and subjected to PCR using primers. The band was evaluated for amounts of mismatch-digestion fragments. To verify the genotype of mice, PCR amplification was performed using AmpliTaqGold®360Mastert Mix (catalog no. 4398881; Thermo Fischer Scientific) from mouse DNA for sequencing. PCR primers were as follows: forward, 5′-ATA GAA CAG TCT AGC CGT AAC TCA C-3′; reverse, 5′-TTC CCA TCT TGG CTG CAT CAC-3′.

Genome DNA was extracted from mouse tails using a DNeasy Blood & Tissue Kit (Cat. no. 69506; Qiagen, Hilden, Germany). The primer sequence used to amplify exon 4 sequences by AmpliTaq Gold^TM^360Master Mix (no. 4398881; Thermo Fisher Scientific) was designed as follows: forward, 5′-TCA TGG ACA CTG AAA TAC AGG-3′; reverse, 5′- AAT AGC GTC AAT GGT CTA AGA G-3′.

Cycling conditions consisted of an initial 12-min denaturation step at 95 °C followed by 35 cycles of 95 °C for 30 s, annealing at 60 °C for 30 s and extension at 72 °C for 30 s, and then final extension at 72 °C for 7 min.

PCR products were sequenced on an ABI 3500 Genetic Analyzer using the BigDye Terminator Cycles Sequencing Kit v3.1 (Thermo Fisher Scientific). Samples were analyzed using Seq Scanner version 2 (Thermo Fisher Scientific) and compared with the public sequence in GenBank (NC_000080.6: *Ptpn20*).

#### Transmission electron microscopy of *Ptpn20****-***knockout mice

8-week-old mice were sacrificed after fixation with 2% paraformaldehyde and 2% glutaraldehyde in 0.1 M phosphate buffer (pH 7.4) administered intravascularly. Cerebellum was removed and brain was divided into two hemispheres. Hemispheres was fixed with 2.5% glutaraldehyde solution (TAAB laboratories Equipment Ltd., Berks, England) in 0.1 M phosphate buffer (pH 7.4), followed by post fixation with 2% OsO4 in the same buffer.

For transmission electron microscopy (TEM), hemispheres were cut into 1 mm-thick axial sections before dehydration. Specimens were dehydrated with a graded series of ethanol, and embedded in Epok812 (Okenshoji Co. Ltd., Tokyo, Japan). Ultrathin sections were cut and stained with uranyl acetate and lead citrate. These sections were examined with an HT7700 transmission electron microscope (Hitachi, Tokyo, Japan).

#### Measurement of the lateral ventricle

Fresh brains 8-week-old WT and *Ptpn20*^*−/−*^ mice were postfixed in 4% paraformaldehyde for 72-h, equilibrated in 30% sucrose solution for 24-h at 4 °C, and then embedded in embedding matrix and frozen at − 80 °C for further use. Coronal sections of 30 μm were obtained using a freezing microtome and stained with HE according to standard methods. We viewed and photographed sections using the E800 microscope, and images were captured with an AxioCam 506 color Digital Camera using AxioVison Rel version 4.7.2.0 image-processing software.

Widths of the brain and lateral ventricles were measured on sections at 0.5 cm anterior to the bregma. The ratio of ventricular width to brain width (VBR) was used to determine the degree of ventricular dilatation [21]. Data were calculated as mean ± standard deviation for WT. Differences between WT and *Ptpn20*^*−/−*^ mice were evaluated using the Mann–Whitney *U* test.

#### Ventricular injection of dye

Eight-week-old WT and *Ptpn20*^*−/−*^ mice were deeply anesthetized by pentobarbital sodium (100 mg/kg; Kyoritsu Seiyaku, Tokyo, Japan). The head was fixed into a stereotaxic apparatus (Narishige Scientific Instrument Laboratory, Tokyo, Japan), and a small burr hole was drilled in the cranium on the right, 1 mm lateral to the bregma. The needle of a microsyringe (MS-10; Ito Corporation, Shizuoka, Japan) was slowly inserted into the lateral ventricle 4 mm from the brain surface through the hole and Evans blue dye (6 µl; FUJIFILM, Wako Pure Chemical Corporation, Osaka, Japan) was injected at 2 µl/min. Ten minutes after injection, mouse brains were harvested, fixed in 4% paraformaldehyde for 72-h, and then cut into 1-mm-thick coronal slices.

#### Real-time PCR, immunoblot and immunofluorescence

These three methods are similar to those used in the first part, but the results in the second part of the real-time PCR were quantified using the 2^−ΔΔCt^ method. Reagents are listed in Additional file 5: Table S2, Additional file 6: Table S3, and Additional file 7: Table S4.

### Statistical analysis

All experimental results are presented as mean ± standard deviation. Data distribution was evaluated graphically using histograms and Q–Q plots. The Shapiro-Wilk test was used to assess the normality of distributions. For the real-time PCR performed as at least five independent experiments, significant differences among groups were determined using Welch’s t-test. Statistical differences of the lateral ventricle between WT and *Ptpn20*^*−/−*^ mice were assessed by the Mann–Whitney U test. All calculations were performed using Prism 9 (GraphPad Software, Inc), with statistical significance set at P < 0.05.

## Results

### Identification of causative gene for hydrocephalus in H-Tx rats

**Fig. 1:**
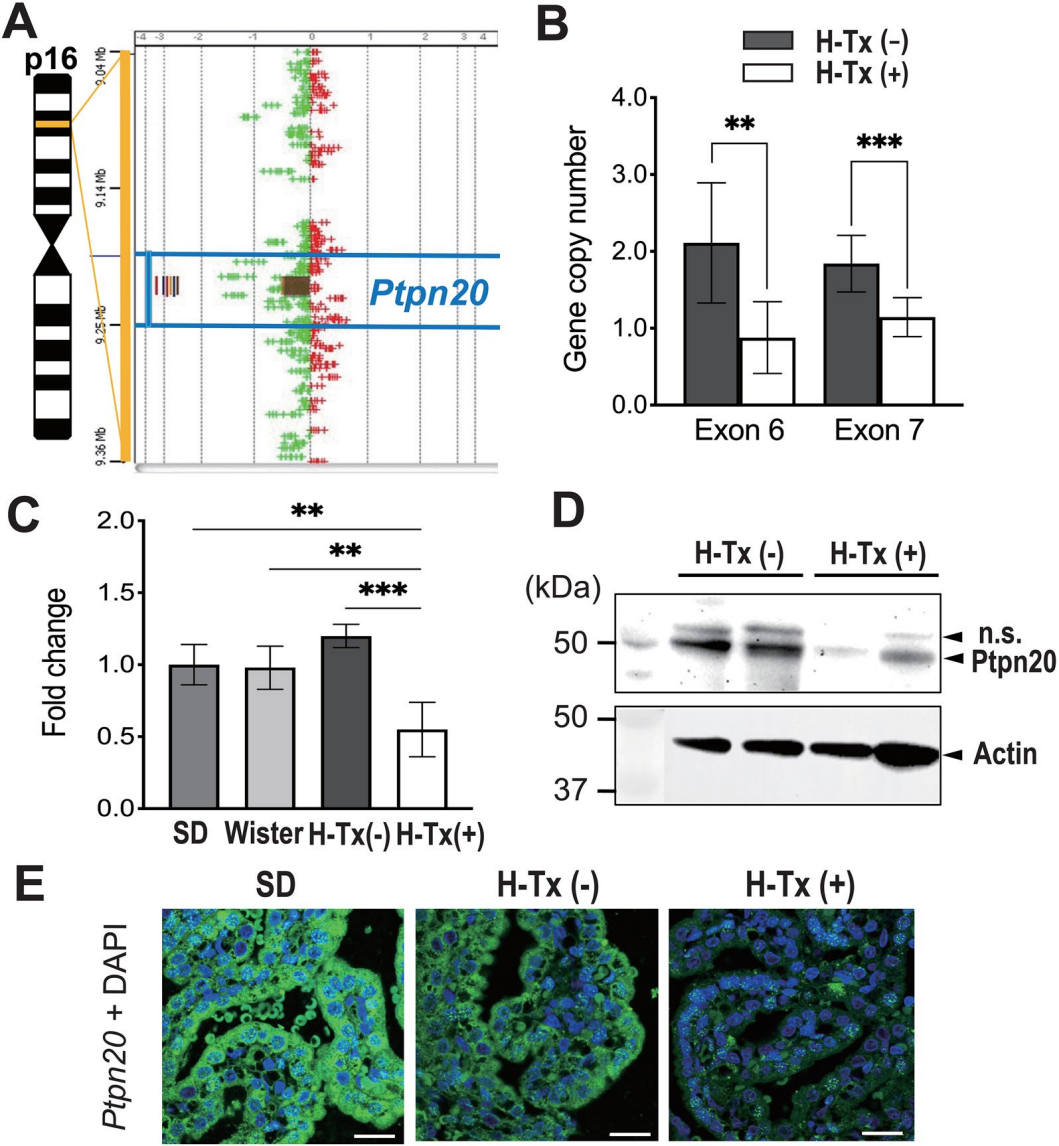
Identification and expression analysis of causative gene in H-Tx rats. **A** The log2 ratio value is plotted on the x-axis. The y-axis represents the genomic position of probes. Genomic profiles indicate a 14-kbp focal loss in 16p16. Colored vertical lines indicate the number of samples with copy number loss and loss segments (brown square) with respect to *Ptpn20*. **B** Real-time PCR of copy number in the *Ptpn20* exon of H-Tx (−) and H-Tx (+) rats at E18. exon 6: H-Tx (−) = 2.11 ± 0.78, H-Tx (+) = 0.88 ± 0.47; exon 7: H-Tx (−) = 1.84 ± 0.37, H-Tx (+) = 1.15 ± 0.25 (mean ± standard deviation, **P < 0.01, ***P < 0.001; H-Tx (−) rats, n = 12; H-Tx (+) rats, n = 6). **C** Real-time PCR of RNAs from P1 rats in SD, Wistar, H-Tx (−) and H-Tx (+) groups to determine expression levels of *Ptpn20* in the brain. Data are normalized to β-actin and fold change is expressed relative to levels in the SD rat. SD = 1.00 ± 0.14; Wistar = 0.98 ± 0.15; H-Tx (−) = 1.20 ± 0.08; H-Tx (+) = 0.55 ± 0.19 (mean ± standard deviation, **P < 0.01, ***P < 0.001; n = 5 rats per group). **D** Immunoblot analysis of *Ptpn20* protein from the CP of P1 H-Tx (−) and H-Tx (+) rats. The 50-kDa bands correspond to *Ptpn20* and nonspecific (n.s.) bands, respectively. *Ptpn20* protein shows low or approximately abolished expression in H-Tx (+) rats. Actin was used as a loading control (n = 5 rats per group). **E** Immunofluorescence images of the CP in P1 SD, H-Tx (−) and H-Tx (+) rats are immunolabeled with anti-*Ptpn20* (green) antibody and with DAPI (blue). *Ptpn20* appears enriched in the CP epithelium of SD and H-Tx (−) rats, with low expression in H-Tx (+) rats, Scale bar = 20 μm

The SurePrint G3 Rat Genome CGH Microarray kit was used to compare the copy numbers of genes in the brains of non-hydrocephalic H-Tx (H-Tx (−)) rats and hydrocephalic H-Tx (H-Tx (+)) rats. Among 30,584 genes tested, we found 47 genes with copy number variations in the brains. Although we did not detect any copy number variations that were present in all H-Tx (+) rats and not in H-Tx (−) rats among these 47 genes, a 14-kbp loss from chromosome region 16p16 was found in all four H-Tx (+) rats (copy number loss), and six of eight H-Tx (−) rats showed no copy number loss encompassing the *Ptpn20* gene (Fig. 1A, Additional file 4: Table S1). We further confirmed and quantified the alteration of copy numbers for the *Ptpn20* exon in twelve H-Tx (−) rats and six H-Tx (+) rats by real-time polymerase chain reaction (PCR). H-Tx (+) rats exhibited an approximately 2.4-fold decrease in exon 6 (P < 0.01) and 1.6-fold decrease in exon 7 (P < 0.001) in copy numbers of *Ptpn20* relative to H-Tx (−) rats (Fig. 1A). After this, we confirmed differential expression of *Ptpn20* mRNA in the brains of H-Tx (+) rats, H-Tx (−) rats, SD rats and Wistar rats by real-time PCR. H-Tx (+) rats showed almost half the amount of *Ptpn20* mRNA expression compared to other rat lineages and H-Tx (−) rats (Fig. 1B). Subsequently, we evaluated *Ptpn20* protein expression in H-Tx (−) and H-Tx (+) rats using immunoblot, which showed immunoreactivity to a 50-kDa *Ptpn20* protein to be approximately absent or lower in the CP of H-Tx (+) rats (Fig. 1C). Moreover, immunofluorescent staining (IF) also showed *Ptpn20* immunoreactivity in CP epithelial cells of H-Tx (−) and SD rats, while immunoreactivity was low in H-Tx (+) rats (Fig. 1D).

### Analysis of *Ptpn20* expression in mice

**Fig. 2:**
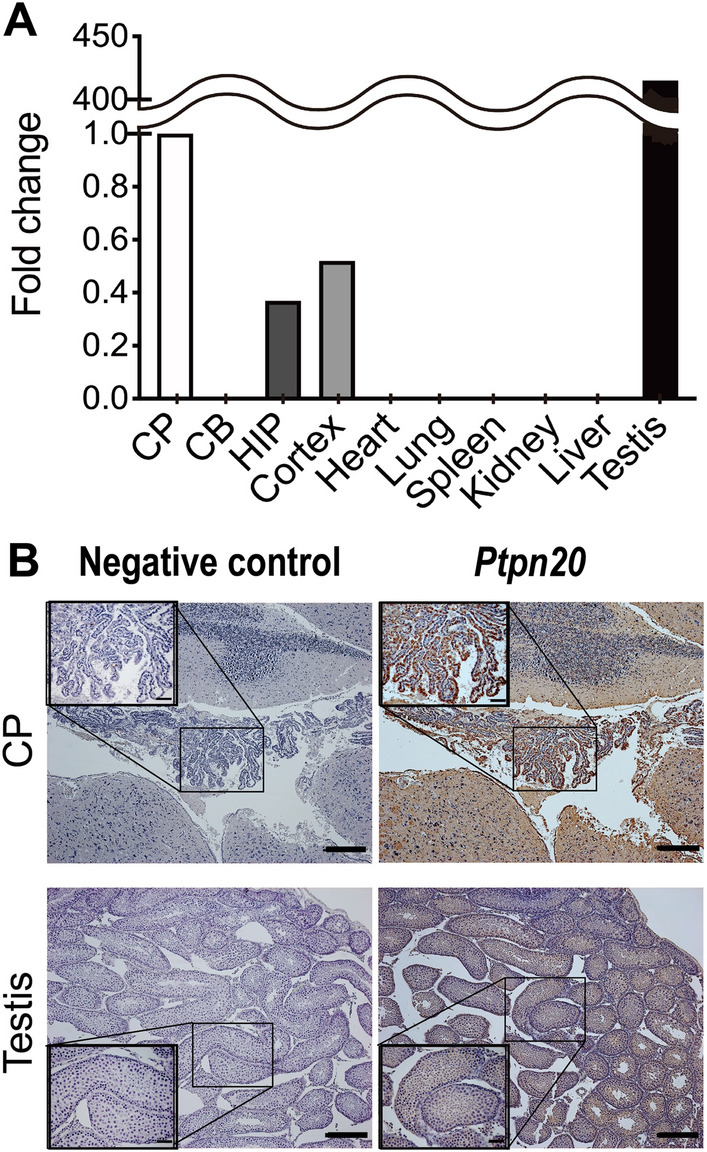
*Ptpn20* expression in mice. **A** *Ptpn20* mRNA expression level in different brain regions and several tissues of 4-week-old C57BL/6J mice as analyzed by real-time PCR. Relative fold change was calculated using the 2^−ΔΔCt^ method. Data are normalized to β-actin and fold change is expressed relative to levels in the CP set at 1. Other values are as follows: hippocampus = 0.37; cortex = 0.52; testis = 415 (hippocampus = HIP, cerebellum = CB; n = 5 mice per group). **B** Representative photomicrographs of immunohistochemistry staining with anti-*Ptpn20* antibody in the CP of the third ventricle and testis from 4-week-old C57BL/6J mice. Scale bar = 50 μm; for zoom = 20 μm

To assess *Ptpn20* expression and distribution in C57BL/6J mice, we examined the expression of *Ptpn20* mRNA in different regions of the brain as well as several tissues (heart, lung, spleen, kidney, liver, and testis) from 4-week-old mice using real-time PCR. *Ptpn20* mRNA was expressed mainly in brain and testis. In the brain, *Ptpn20* mRNA was expressed in the CP, hippocampus, and cerebral cortex. *Ptpn20* mRNA expression levels were 2.73- and 1.92-fold higher in the CP than in the cerebral cortex and hippocampus, respectively. However, *Ptpn20* mRNA showed its highest expression in the testis, at 415-fold higher than in the CP. In contrast, no expression was detected in other tissues (Fig. 2A). These results were further validated by immunohistochemical staining of murine heart, lung, spleen, pancreas, liver, testis, and CP using *Ptpn20* antibody. The staining showed extremely strong immunoreactivity in the testis and CP epithelial cells, but no change in other tissues (Fig. 2B, Additional file 2: Fig. S2).

### Generation of *Ptpn20*-knockout mice

**Fig. 3:**
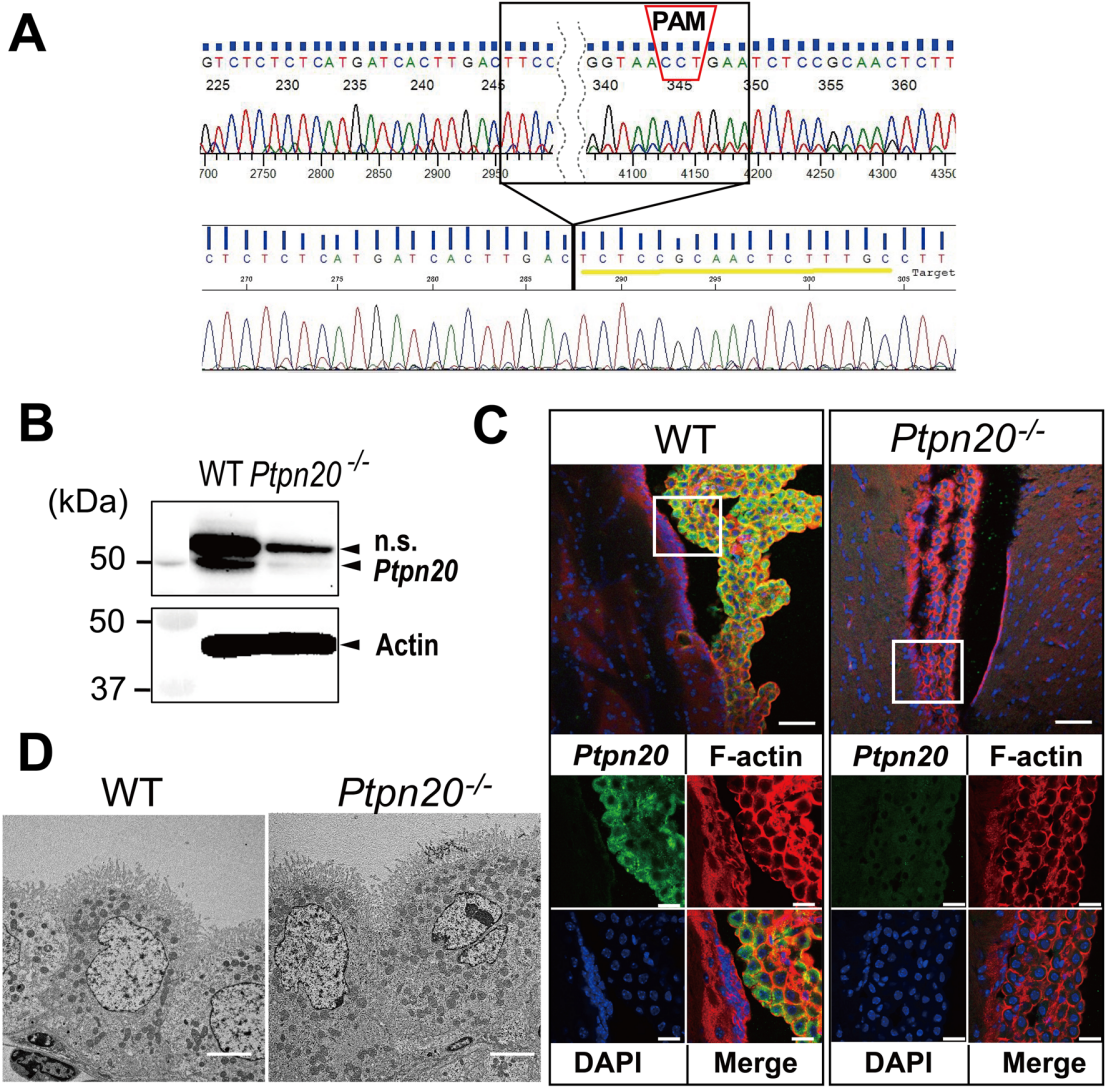
Generation of *Ptpn20*^−/−^ mice. **A** Schematic illustration of *Ptpn20* locus. *Ptpn20* gene was targeted at exon 4. The sgRNA sequence is underlined in yellow. The protospacer adjacent motif sequence is indicated in the red dotted box. Electropherograms of the 104-bp deletion mutations in exon 4 are indicated in the black dotted box (sgRNA = single guide RNA). **B** Expression levels of *Ptpn20* protein in CP were verified by immunoblotting. The 50-kDa bands correspond to Ptpn20 and nonspecific bands. *Ptpn20* protein shows almost completely absence in the CP of *Ptpn20*^*−/−*^ mice. Actin is used as the loading control (n = 5 mice per group). **C** Immunofluorescence of *Ptpn20* (green) and F-actin (red) in the CP of 8-week-old WT and *Ptpn20*^*−/−*^ mice. *Ptpn20* is almost completely absent in the CP of *Ptpn20*^−/−^ mice, while no significant difference is evident for F-actin compared to WT. Areas in square frames are magnified in the bottom panels. Top panels, scale bar = 50 μm; bottom panels, scale bar = 20 μm. **D** Representative transmission electron microscope images of longitudinal sections of lateral ventricle CP from 8-week-old WT and *Ptpn20*^*−/−*^ *mice.* No structural difference in CP is evident between WT and *Ptpn20*^*−/−*^ mice. Scale bar = 20 μm

To investigate whether deletion of *Ptpn20* represents a risk factor for hydrocephalus, we generated *Ptpn20*-knockout (*Ptpn20*^*−/−*^) mice using CRISPR/Cas9 technology. We targeted and generated a sgRNA against exon 4 of the *Ptpn20* mouse gene. DNA sequencing results confirmed the targeted 104-bp deletion mutations in *Ptpn20*^*−/−*^ mice (Fig. 3A). For immunoblot analysis, *Ptpn20* protein was almost completely absent in the CP of *Ptpn20*^*−/−*^ mice (Fig. 3B). We then performed IF using anti-*Ptpn20* and anti-F-actin antibodies. *Ptpn20* was detected in the cytoplasm of CP epithelial cells in WT mice, but not in ependymal cells, and expression was completely abolished in *Ptpn20*^*−/−*^ mice. F-actin staining showed no obvious difference between WT and *Ptpn20*^*−/−*^ mice (Fig. 3C). Transmission electron microscopy (TEM) applied to detect substantial structural differences revealed no obvious differences between the CP epithelial cells of WT and *Ptpn20*^*−/−*^ mice (Fig. 3D).

### *Ptpn20*^*−/−*^ mice develop communicating hydrocephalus

**Fig. 4:**
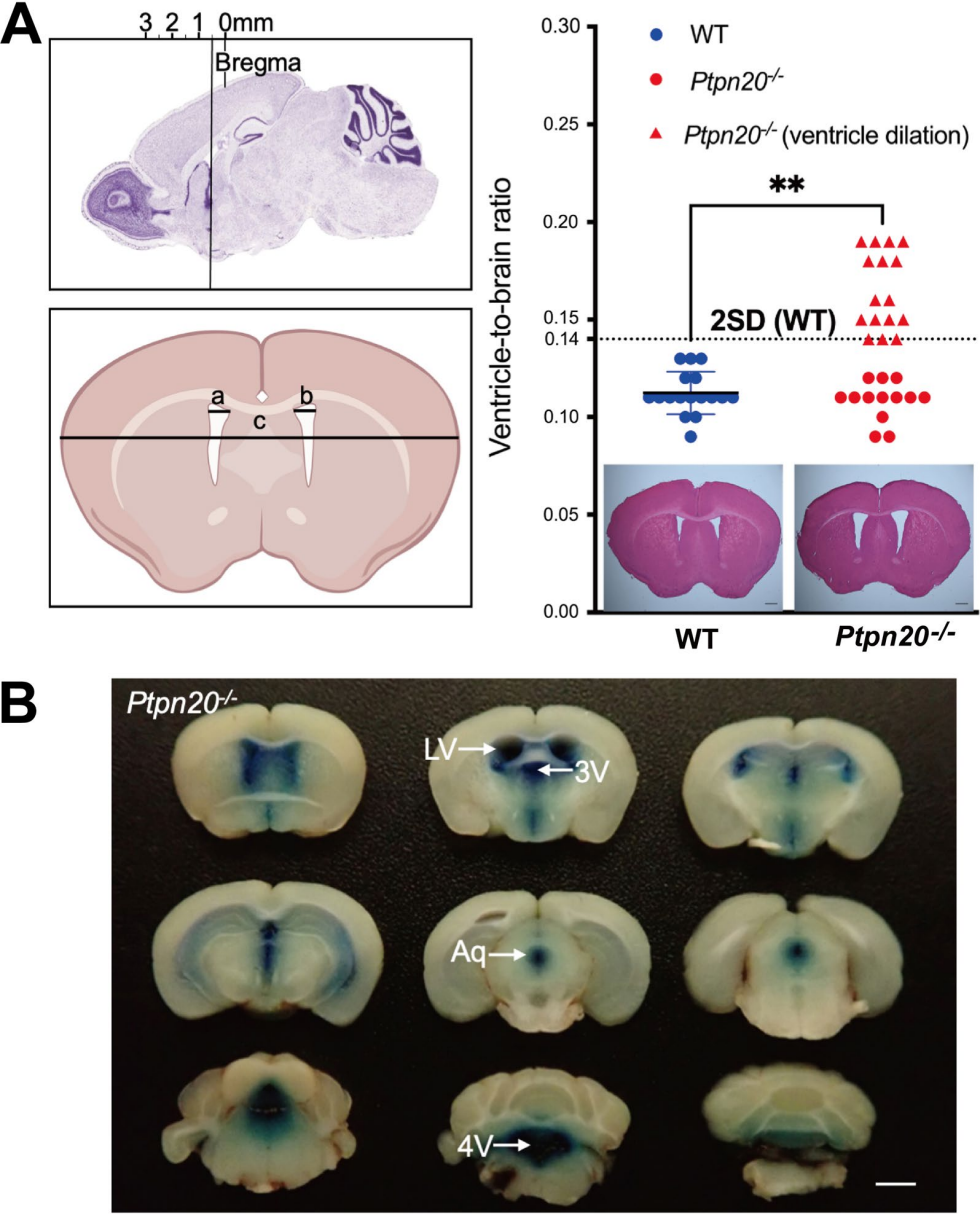
*Ptpn20*^*−/−*^ mice develop communicating hydrocephalus. **A** Sagittal brain section showing acquisition sites of coronal brain slices. Representative micrograph of coronal and HE-stained brain sections showing distances measured for ventricle width (left, a; right, b) and brain width (c) in WT and *Ptpn20*^−/−^ mice. Ventricle-to-brain ratio (VBR) is calculated as the ratio of the sum of ventricular widths to brain width ((a + b)/c). *Ptpn20*^−/−^ mouse brain shows dilation of lateral ventricles. Histograms showing the VBR of WT and *Ptpn20*^−/−^ mice (mean ± standard deviation, Mann-Whitney U test, **P < 0.01; WT, n = 17; *Ptpn20*^−/−^ mice, n = 29). **B** Evans Blue injected into the lateral ventricles of *Ptpn20*^−/−^ mice at 8 weeks old shows slight enlargement of lateral ventricles with dye also seen in the fourth ventricle (n = 5; LV = lateral ventricle; 3V = third ventricle; Aq = aqueduct; 4V = fourth ventricle). Scale bar = 2 mm

*Ptpn20*^*−/−*^ mice seemed to have normal appearance and lifespan, because almost all lived to over 18 months. To elucidate the morphology of the brain and ventricles of *Ptpn20*^*−/−*^ mice, we analyzed brain slices from 8-week-old mice by hematoxylin and eosin (HE)-staining. Mean relative ventricle-to-brain ratio (VBR) was 0.11 ± 0.01 for WT mice and 0.14 for *Ptpn20*^−/−^ mice (P < 0.01). In *Ptpn20*^−/−^ mice, VBR exceeding the mean WT value by 2 standard deviations was defined as indicating hydrocephalus. Of these 29 *Ptpn20*^−/−^ mice, 16 mice met the criteria for hydrocephalus, and 13 mice did not develop hydrocephalus, while some of the hydrocephalic mice exhibited very mild ventriculomegaly (Fig. 4A). To establish whether an anatomical defect resulted in hydrocephalus, we injected Evans blue in the lateral ventricle of *Ptpn20*^−/−^ mice. Blue dye appeared in the aqueduct, the fourth ventricle and the atlantooccipital subarachnoid space in all cases, suggesting the entire CSF drainage pathway was patent (Fig. 4B).

### Phosphorylation of NKCC1 is maintained in CP epithelial cells of *Ptpn20*^*−/−*^ mice

**Fig. 5:**
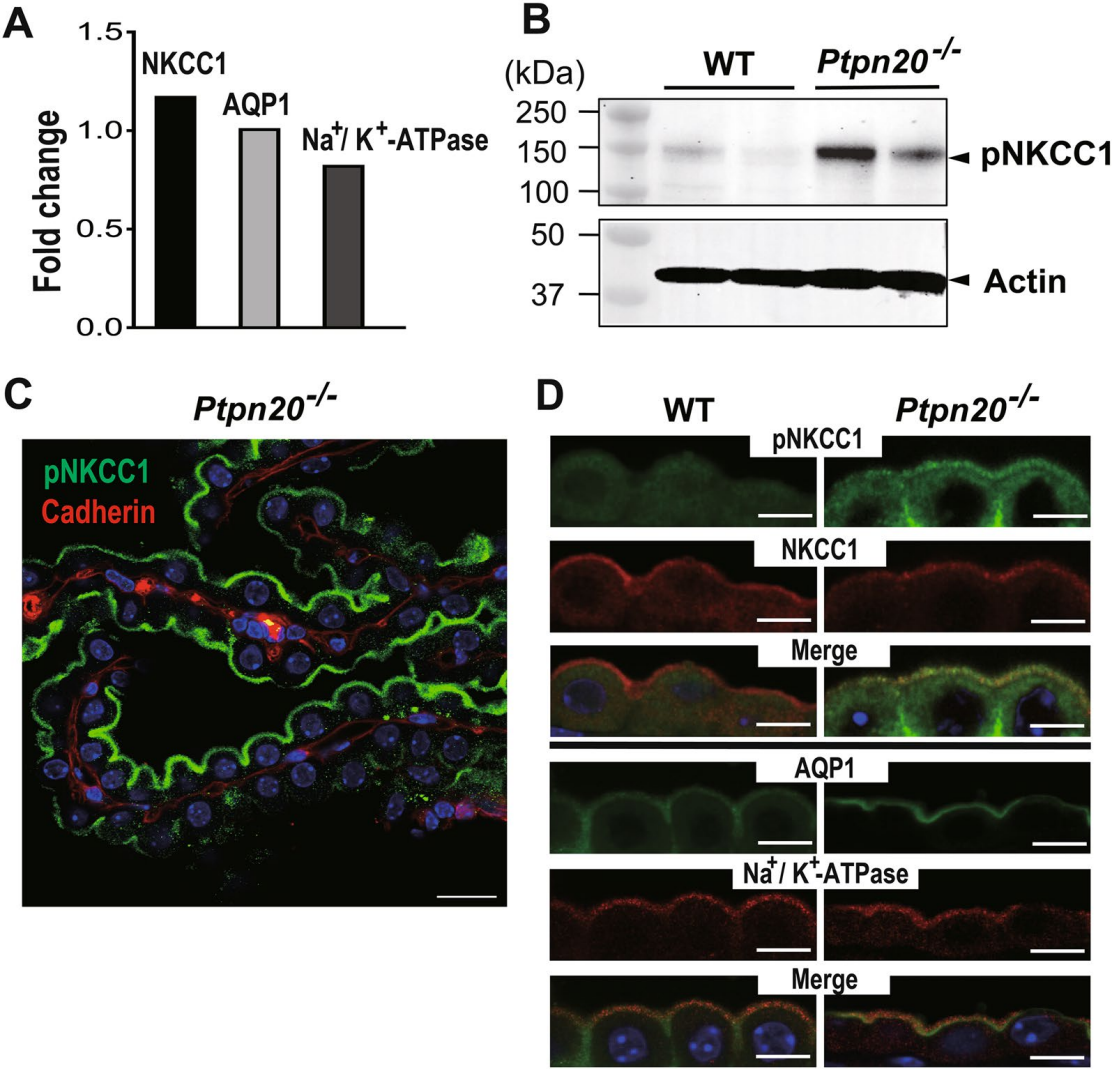
pNKCC1 expression is increased in the CP of *Ptpn20*^*−/−*^ mice. **A** Expressions of *Ptpn20*, water and ion transporters in the CP of *Ptpn20*^*−/−*^ mice as analyzed by real-time PCR. Relative fold change is calculated using the 2^−ΔΔCt^ method. Data are normalized to β-actin and fold change is expressed relative to the expression level of *Ptpn20* in the CP of WT mice set as 1. We did not find significant differences in expressions of NKCC1, AQP1 or Na^+^/K^+^-ATPase in CP between 8-week-old WT and *Ptpn20*^*−/−*^ mice. Values are as follows: NKCC1 = 1.18; AQP1 = 1.01; ATP1a1 (Na^+^/K^+^-ATPase) = 0.83 (n = 5 mice per group). **B** Immunoblotting of pNKCC1 protein expression in the CP of 8-week-old WT and *Ptpn20*^*−/−*^ mouse. Expression of pNKCC1 is higher in the CP of *Ptpn20*^*−/−*^ mice than in WT mice. Actin is used as the loading control (n = 5 mice per group). **C** Immunofluorescence of *Ptpn20*^*−/−*^ mouse CP illustrating expression of pNKCC1 in the membrane facing the lumen (green) with E-cadherin (red) as a basolateral marker. Scale bar = 20 μm. **D** Immunofluorescence of pNKCC1, NKCC1, AQP1 and Na^+^/K^+^-ATPase in 4-week-old WT and *Ptpn20*^*−/−*^ mice in the CP of the lateral ventricles and fourth ventricle are immunolabeled as shown. *Ptpn20*^*−/−*^ mice show stronger pNKCC1 expression in the cytoplasm and apical surface of CP epithelial cells than WT mice. No significant differences are seen in NKCC1, AQP1 or Na^+^/K^+^-ATPase between WT and *Ptpn20*^*−/−*^ mice. Scale bar = 10 μm

To further explore connections between the pathogenesis of hydrocephalus and CP in *Ptpn20*^−/−^ mice, we compared aquaporin 1 (AQP1), sodium–potassium adenosine triphosphatase (Na^+^/K^+^-ATPase), and Na-K-Cl cotransporter (NKCC1) expressions in the CP, as proteins involved in the secretion or absorption of CSF. No significant differences in the mRNA expressions of AQP1, Na^+^/K^+^-ATPase, or NKCC1 were detected in the CP by real-time PCR (Fig. 5A). We then used immunoblotting techniques to investigate the expression of phosphorylated NKCC1 (pNKCC1) in the CP, which correlates with the phosphorylation of Thr212/Thr217 in its N-terminal domain. *Ptpn20*^−/−^ mice showed differential increase in levels of pNKCC1 expression from 8 weeks until 72 weeks compared with WT (Fig. 5B, Additional file 3: Fig. S3A). Furthermore, consistent with our immunoblotting results, IF analysis of CP sections labeled with pNKCC1 antibody showed significantly increased expression of pNKCC1 in the CP epithelial cells in *Ptpn20*^−/−^ mice and verified the localization of pNKCC1 at the apical membrane (Fig. 5C), while no significant differences were seen there for unphosphorylated NKCC1, AQP1 or Na^+^/K^+^-ATPase compared with the WT (Fig. 5D).

## Discussion

This study identified copy number loss of *Ptpn20* in the brain of H-Tx (+) rats. *Ptpn20* was enriched in the CP of non-hydrocephalic rats, but significantly decreased in the CP of H-Tx (+) rats. We then generated *Ptpn20*-deficient mice to investigate whether the deletion of *Ptpn20* is a risk for hydrocephalus. *Ptpn20*^−/−^ mice did not exhibit the characteristics of congenital hydrocephalus observed in H-Tx rats after birth. However, further analysis in adult *Ptpn20*^−/−^ mice revealed ventriculomegaly in more than half of these mice without significant blockage of CSF circulatory pathways, thus indicating that *Ptpn20*^*−/−*^ mice had developed communicating hydrocephalus. Since hydrocephalus is not associated with stenosis of the cerebral aqueduct, we further investigated the CP, which is mainly affected by *Ptpn20* deletion. Previous studies have shown that structural or functional abnormalities of CP may affect CSF production and thus lead to the development of hydrocephalus [31–33]. To evaluate possible CP abnormalities, we analyzed the ultrastructure of the CPs of the lateral and third ventricles in *Ptpn20*^*−/−*^ mice using TEM. A normal appearance of the CP epithelium and intact junctions between cells suggested that passive diffusion of molecules from the CP vasculature into the CSF is unlikely. However, in analyzing the channels and transporters involved in ion and water transport for CP epithelial cells, we found that expression of pNKCC1 protein was mainly at the apical surface of CP epithelial cells and was significantly increased in about half of *Ptpn20*^−/−^ mice as compared with WT mice. This result may provide useful insights into the pathogenesis of hydrocephalus, and the individual differences in the degree of pNKCC1 expression seem to explain why only half of the knockout mice developed hydrocephalus.

NKCC1 is located on the basolateral membrane and is involved in active transportation of Na^+^, K^+^, and Cl^−^, which play important roles in fluid absorption and secretion in most epithelial tissues. However, NKCC1 in the brain is highly expressed in the apical membrane of CP epithelial cells [34]. Several studies have shown that inhibition of NKCC1 by bumetanide leads to decreased CSF production in the CP [29, 35–37]. Ex vivo and in vivo studies have shown that NKCC1 contributes approximately half of the CSF production that is independent of the osmotic gradient [29]. In addition, previous studies have shown that increased NKCC1 activity is associated with direct phosphorylation of NKCC1 [38], suggesting that enhanced or continued NKCC1 phosphorylation may result in greater CSF production by the CP. This hypothesis is supported also by a rat model of posthemorrhagic hydrocephalus in which inflammation-induced phosphorylation of NKCC1 caused hypersecretion of CSF [39].

*Ptpn20* is a member of the PTPs family, which, together with protein tyrosine kinases, is responsible for regulating the phosphorylation state of intracellular protein [40]. The function of *Ptpn20* is not yet completely understood. Limited studies have shown that *Ptpn20* is expressed in a variety of cell lines, showing a dynamic subcellular distribution in response to various extracellular stimuli targeting sites of actin polymerization [41]. Previous studies have shown that inhibition of actin polymerization with cytochrome D increases NKCC1 activity in colonic cells [42, 43]. However, our results did not show obvious differences in F-actin staining (Fig. 3C), suggesting that *Ptpn20* correlates with actin, but has no significant effects on actin polymerization or depolymerization.

On the other hand, NKCC1 activity is known to be regulated by phosphorylation of its specific Serine/Threonine residues in other cell types, and Ste20/SPS1-related proline/alanine-rich kinase, as an upstream regulator of NKCC1, can phosphorylate the N terminus of NKCC1 at Thr203, Thr207 and Thr212 [44, 45].

It is not clear why NKCC1 can be activated by tyrosine kinase in the absence of tyrosine sites. However, based on this finding, we can hypothesize that NKCC1 may also be dephosphorylated by the protein tyrosine phosphatase encoded by *Ptpn20*. In addition, a portion of intracellular protein tyrosine phosphatases in the nervous system also function as dual specificity phosphatases that can dephosphorylate both phosphotyrosine and phosphoserine or phosphothreonine [46]. That is, if *Ptpn20* also possesses the properties of dual specificity phosphatase, serine or threonine residues that are phosphorylated in NKCC1 and its upstream regulators can also be directly dephosphorylated by *Ptpn20*. Therefore, deletion of *Ptpn20* may thus cause NKCC1 to fail to dephosphorylate and persistently remain in the phosphorylated state, and our results showing increased expression of pNKCC1 in *Ptpn20*^−/−^ mice from 8 to 72 weeks support this concept. This change would result in a continuous excess of CSF entering the ventricular system. In humans, CSF hypersecretion secondary to CP hyperplasia or CP papilloma disturbs the homeostasis of CSF production and absorption in the brain, leading to hydrocephalus [47, 48], and higher secretion rates correlate with more severe hydrocephalus [49]. We therefore consider that CSF hyperproduction may be a direct cause of hydrocephalus in this study. To further confirm this hypothesis, we also examined pNKCC1 expression in H-Tx rats, and we found that pNKCC1 expression was significantly increased in the CP of H-Tx rats compared to SD rats (Additional file 3: Fig. S3B). This result suggests that increased expression of pNKCC1 due to *Ptpn20* deficiency is a potential factor contributing to hydrocephalus in both rats and mice. Notably, the interpretation of ventriculomegaly is complicated by a range of potential causative factors. In addition to CP hypersecretion, which leads to lateral ventricular dilatation, other factors can contribute to hydrocephalus, such as cerebral compliance and arachnoid drainage [50–53].

However, *Ptpn20*^*−/−*^ mice did not exhibit severe hydrocephalus after birth like H-Tx rats, and we hypothesize that this may be because *Ptpn20*^*−/−*^ mice did not develop aqueductal stenosis during development or that the overproduction of CSF caused by NKCC1 in the genetic background of C57BL/6J was insufficient to cause severe hydrocephalus. Aqueductal stenosis is associated with abnormal development and dysfunction of the subcommissural organ (SCO) in H-Tx rats. The SCO is a secretory gland of epithelial cells dorsal to the aqueduct of the brain, and in H-Tx rats with congenital hydrocephalus, immunoreactivity of the SCO and glycoproteins of midbrain epithelial cells is decreased starting from embryonic day 16, before ventricular enlargement. Abnormal secretion of glycoprotein is known to interfere with the formation of Reissner’s fiber, obstructing the cerebral aqueduct and leading to ventricular enlargement on embryonic day 17 [54–57]. This finding is consistent with other animal models regarding the link between SCO function and hydrocephalus [58]. Although the gene for hydrocephalus in H-Tx rats has not been identified, quantitative trait analysis suggests that the loci associated with the hydrocephalus phenotype are present on chromosomes 9, 10, 11, and 17 [23]. In contrast, the *Ptpn20* gene, located on chromosome 16, may not be involved in the developmental process of SCO.

Furthermore, in a study of L1-deficient mice, significantly enlarged ventricles were observed only in mutant mice backcrossed to the C57BL/6J genetic background, whereas the ventricular system appeared normal when the same mutant mouse strain was backcrossed to the 129 genetic background [59]. In addition, Cai et al. [18] found a lower than expected incidence of hydrocephalus and milder disease than in H-Tx rats when they cross-mated hydrocephalic H-Tx rats, and suggested that this result might be due to the effects of gene modifications in different genetic backgrounds. Similarly, the role of the *Ptpn20* gene may be enhanced in the genetic background of H-Tx rats and diminished in C57BL/6J mice. In addition, another study on L1 mutants showed that ventricular enlargement preceded the onset of aqueductal stenosis, and that massive enlargement of the ventricles caused deformation of the brain, which in turn compressed the aqueduct, leading to severe hydrocephalus [60]. Based on this hypothesis, we consider that although phosphorylation of NKCC1 may produce excess CSF, its production rate might be relatively mild and constant, and so may be insufficient to create an unbalanced pressure differential within the ventricular system that would result in brain deformation. As a result, only moderate hydrocephalus developed.

The interpretation of ventriculomegaly is complicated by a range of potential causative factors. While excessive CP secretion undoubtedly leads to lateral ventricular dilatation, other factors can also contribute to ventriculomegaly, such as cerebral compliance and arachnoid drainage, both acute and chronic. NKCC1 is also expressed in the arachnoid and is likely involved in fluid formation in arachnoid cysts [61, 62]. In the current study we did not evaluate NKCC1 expression on the arachnoid. However, if the hypothesis about NKCC1 is reasonable, it is also worthwhile to investigate in depth whether *Ptpn20* deletion causes functional alterations in arachnoid NKCC1. This is because it is possible that arachnoid NKCC1-mediated changes in water and osmolarity could affect the absorptive function of arachnoid granules or cause an increase in subarachnoid water production. Whether this is also responsible for the chronic state of hydrocephalus in H-Tx rats also requires further investigation.

As previously mentioned, the direction of NKCC1 transport has been a controversial topic. As proposed by Steffensen et al., CP apical NKCC1 works in a net efflux mode, co-transporting ions and water and directly contributes to the continued production of CSF [29]. Gregoriades et al. objected that under basal conditions CP apical NKCC1 works in a net instream mode, transporting ions and associated water into the cell, maintaining intracellular Cl− concentrations and volume of cytosolic water required for CSF secretion [28]. However, Steffensen’s group refuted Gregoriades et al. by suggesting that the results they derived for the direction of NKCC1 endocytic transport may stem from the treatment of the cells prior to the experiment and the contents of the solutions tested during the experiment [63]. As a further basis for the inward NKCC1 transport pattern, Xu et al. found that CP NKCC1 mediates the clearance of CSF during early postnatal development in mice, and in addition, it was found that overexpression of CP NKCC1 in a model of postnatal obstructive hydrocephalus resulted in a reduction in ventricular enlargement [30]. It has to be said that this finding provides additional strong evidence for the inward transport of CSF at a particular stage in mice. However, for the current study, this inward transport mechanism is inconsistent with our latest findings, and in the absence of other factors contributing to ventricular enlargement, outward transport of CSF by CP NKCC1 is the most plausible explanation at this stage.

About 100 years ago the CP was thought to be the main site of CSF production, and since then choroid plexus cauterization (CPC) has been developed to reduce CSF production for the treatment of hydrocephalus. In the early twentieth century, CPC alone was used with some success in patients with communicating hydrocephalus, but it was abandoned later due to the high mortality rate and the poor outcome observed [64–66]. With the advance in neurosurgical techniques, CPC has received renewed attention, but is mostly used as an adjunct to endoscopic third ventriculostomy (ETV) in the treatment of hydrocephalus [67–71]. Although it seems that CPC alone has become an obsolete procedure, a question remains if there is still a value of CPC in the treatment of any specific form of hydrocephalus. ETV + CPC is reported to be effective in pediatric hydrocephalus.　Many of the reported patients were considered successful because intracranial pressure was controlled during the acute phase and shunt placement was not necessary, however detailed evaluation of psychomotor development was not performed. Therefore currently, the indicated cases are considered to be children with extremely high rates of shunt dysfunction and patients with hydranencephaly, in whom future psychomotor development is not expected [72]. The rationale for the success of ETV + CPC is the reduction of intraventricular pulsatility due to the CP, in addition to the reduction of CP CSF production [73]. The confirmation of this consideration requires accumulation of basic research on intraventricular pulsatility [74]. However, we must note that other potential functions of the CP besides CSF secretion should not be neglected. In particular, NKCC1, the focus of attention in this study, has been shown to play a key role in regulating cell volume and maintaining ionic homeostasis in the CNS [75, 76]. Furthermore, the CSF produced from the CP is also thought to play additional role. The CP releases various hormones, cytokines, and other proteins as CSF, which act on the thalamus, hypothalamus, periventricular organs (pineal gland), and some neuronal nuclei (suprachiasmatic nucleus).In that way it plays an important role in maintaining homeostasis, including autonomic daily rhythm, emotion, and stress response [77, 78]. Furthermore, the role of amyloid-β elimination in the CSF has attracted attention in relation to the pathogenesis of Alzheimer’s disease, and it has been reported that amyloid-β metabolizing enzyme system proteins are released from the CP in conjunction with the circadian rhythm and that amyloid-β is absorbed directly into the blood from the CP [79].

This study had several limitations. First, since accurately determining whether H-Tx rats at P18 have hydrocephalus based solely on appearance is difficult, hydrocephalic H-Tx rats may have been present among the current non-hydrocephalus samples, masking other genetic risk factors. Future studies will be needed, with larger sample sizes and improved sensitivity of the results, to identify other possible causative genes. Eventually, paired or multiple knockout models can be established to further clarify the molecular mechanisms underlying hydrocephalus. Second, since reliable methods to accurately measure CSF production remain lacking, it is not possible to test the hypothesis of CSF overproduction in *Ptpn20*^*−/−*^ mice. Third, we focused primarily on changes in membrane transport proteins located on the apical side of the CP epithelium, however, Na^+^-driven chloride bicarbonate exchanger located on the basolateral side of the epithelium also plays a role in CSF production. Whether *Ptpn20* deletion leads to any functional changes on the basolateral side warrants further investigation. In addition, the lack of imaging tools in our study makes the degree of ventricular dilatation impossible to determine directly on live animals. Isolated or dehydrated brain tissue may result in varying degrees of morphological and structural alteration of the ventricles, which may in term have affected the accuracy of our results.

## Conclusions

Our strategy of identifying potential pathogenic genes in hydrocephalic H-Tx rats and elucidating mechanisms in transgenic mouse models revealed the unexpected pathological relevance of hydrocephalus to *Ptpn20* deficiency. *Ptpn20* makes a substantial contribution to CSF production and provides a novel approach for diagnosing hydrocephalus. Furthermore, inhibition of the phosphorylation pathway of NKCC1 by protein tyrosine phosphatase may represent a therapeutic target for the management of hydrocephalus in the future.

## Supplementary Information

**Additionalfile 1: Figure S1.** H-Tx rats for genetic risk identification.

**Additionalfile 2: Figure S2.** *Ptpn20* expression in the tissues of mice.

**Additionalfile 3: Figure S3.** Immunoblotting of pNKCC1 protein expression in aged*Ptpn20*^*−/−*^ mice and H-Tx rats.

**Additional file 4: Table S1.** Copy number variations.

**Additional file 5: Table S2.** Assay of real-time PCR.

**Additional file 6: Table S3.** Primary antibodies forimmunoblot investigations.

**Additional file 7: Table S4.** Reagents forimmunofluorescence investigations.

## Data Availability

Data are available from the corresponding author with reasonable request.

## Abbreviations

H-Tx: Hydrocephalus-Texas
H-Tx (−): Non-hydrocephalic H-Tx
H-Tx (+): Hydrocephalic H-Tx
CSF: Cerebrospinal fluid
SD: Sprague-Dawley
WT: Wild type
CGH: Comparative genomic hybridization array
PBS: Phosphate buffered saline
PCR: Polymerase chain reaction
CRISPR: Clustered regularly interspaced short palindromic repeats
sgRNA: Single guide RNA
CP: Choroid plexus
CNS: Central nervous system
IF: Immunofluorescent staining
TEM: Transmission electron microscopy
HE: Hematoxylin and eosin
VBR: Ventricle-to-brain ratio
AQP1: Aquaporin 1
Na^+^/K^+^-ATPase: Sodium–potassium adenosine triphosphatase
NKCC1: Na-K-Cl cotransporter
pNKCC1: Phosphorylated NKCC1
CPC: Choroid plexus cauterization
ETV: Endoscopic third ventriculostomy
