# Supplementary material for: Ptpn20 deletion in H-Tx rats enhances phosphorylation of the NKCC1 cotransporter in the choroid plexus: an evidence of genetic risk for hydrocephalus in an experimental study
Source: Fluids Barriers CNS. 2022 Jun 3;19:39. doi: 10.1186/s12987-022-00341-z (PMC9164390; doi:10.1186/s12987-022-00341-z)
Supplement: Supplementary file 1 — Additionalfile 1: Figure S1. H-Tx rats for genetic risk identification. [file 12987_2022_341_MOESM1_ESM.docx]

| 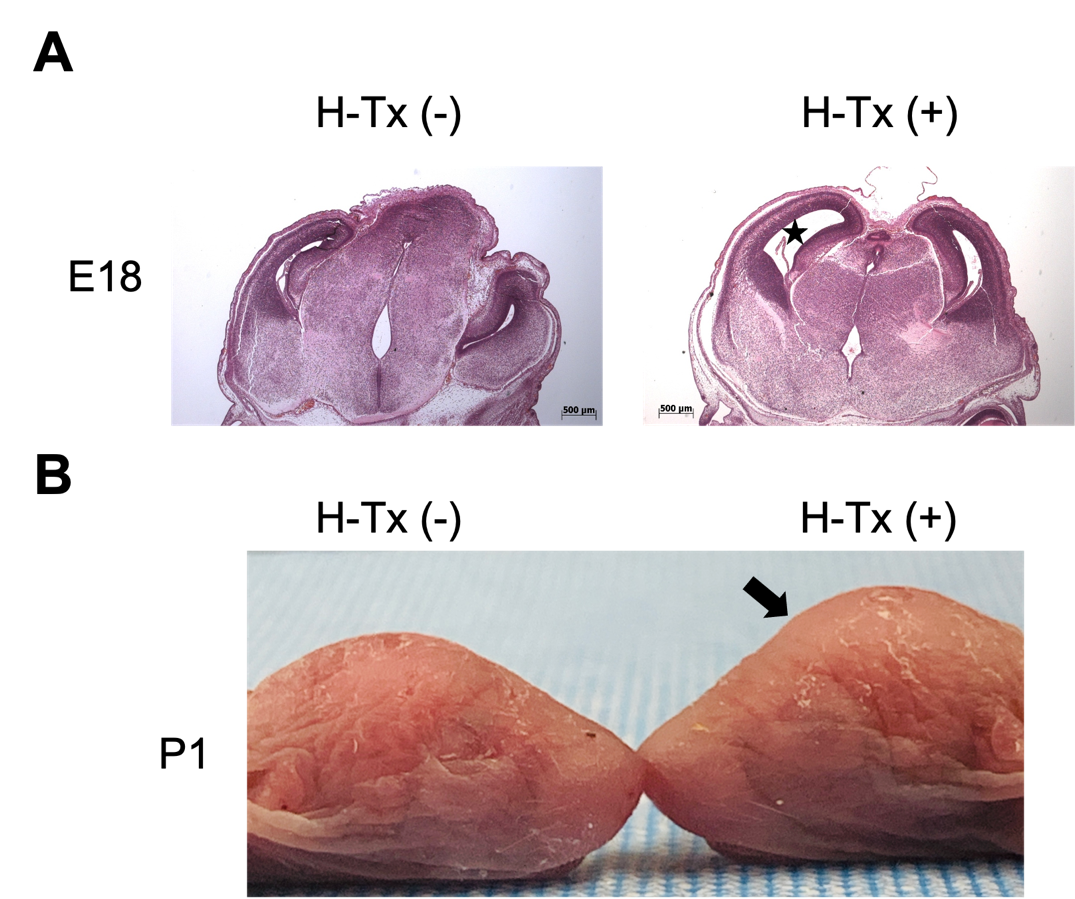 |
| --- |
| **Figure S1. H-Tx rats for genetic risk identification.**  A. HE-stained coronal sections of H-Tx (-) and H-Tx (+) rats at E18, asterisk shows the dilated lateral ventricle. Scale bar = 500 µm.  B. Images of P1 H-Tx (-) and H-Tx (+) rats, arrow points to the domed head. |
