# Supplementary material for: Ptpn20 deletion in H-Tx rats enhances phosphorylation of the NKCC1 cotransporter in the choroid plexus: an evidence of genetic risk for hydrocephalus in an experimental study
Source: Fluids Barriers CNS. 2022 Jun 3;19:39. doi: 10.1186/s12987-022-00341-z (PMC9164390; doi:10.1186/s12987-022-00341-z)
Supplement: Supplementary file 2 — Additionalfile 2: Figure S2. Ptpn20 expression in the tissues of mice. [file 12987_2022_341_MOESM2_ESM.docx]

| 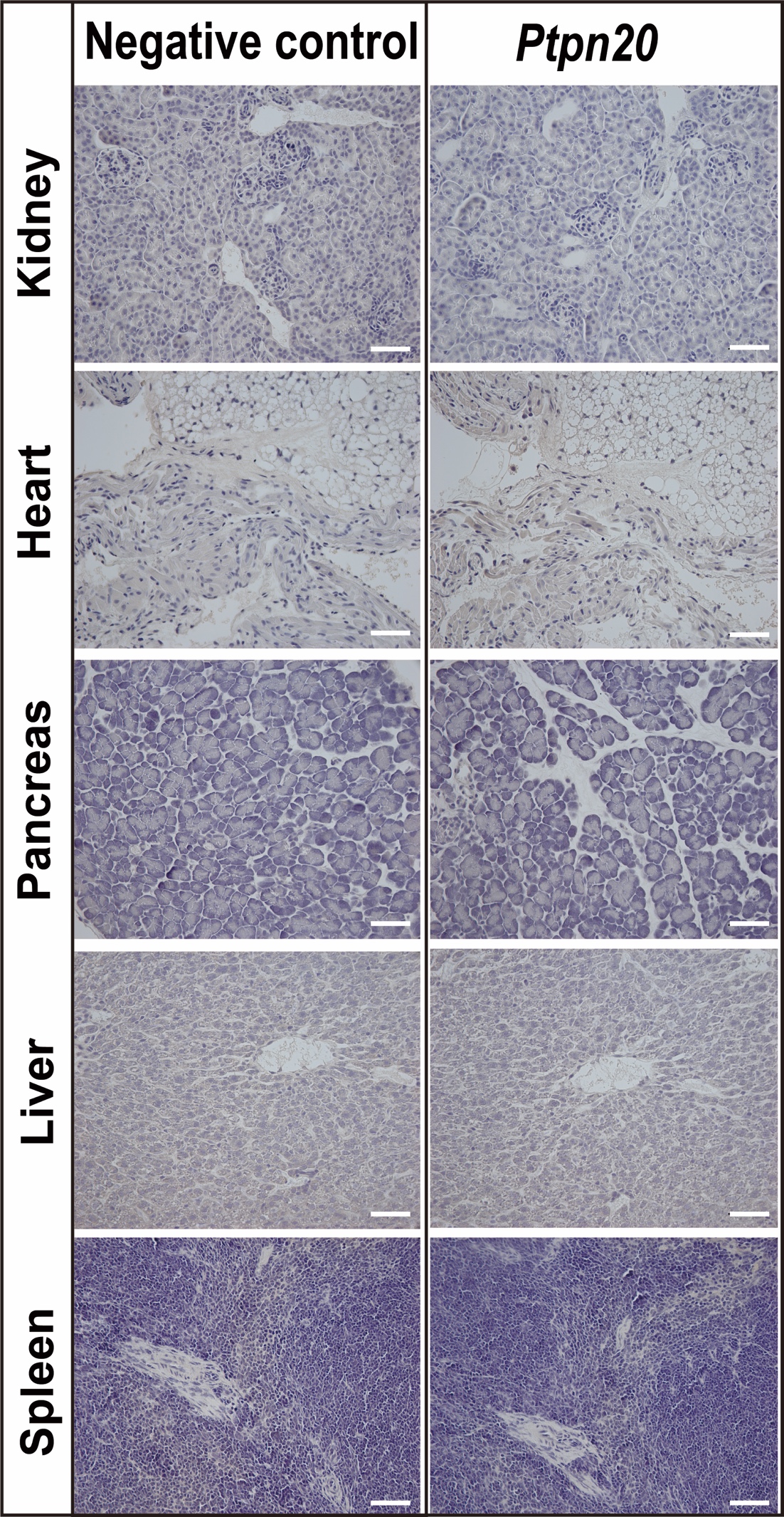 |
| --- |
| **Figure S2. *Ptpn20* expression in the tissues of mice.**  Representative photomicrographs of immunohistochemistry staining with anti-*Ptpn20* antibody in the heart, lung, spleen, pancreas, and liver from 4-week-old C57BL/6J mice. Scale bar = 50 µm. |
