# Supplementary material for: Ptpn20 deletion in H-Tx rats enhances phosphorylation of the NKCC1 cotransporter in the choroid plexus: an evidence of genetic risk for hydrocephalus in an experimental study
Source: Fluids Barriers CNS. 2022 Jun 3;19:39. doi: 10.1186/s12987-022-00341-z (PMC9164390; doi:10.1186/s12987-022-00341-z)
Supplement: Supplementary file 3 — Additionalfile 3: Figure S3. Immunoblotting of pNKCC1 protein expression in agedPtpn20−/− mice and H-Tx rats. [file 12987_2022_341_MOESM3_ESM.docx]

| 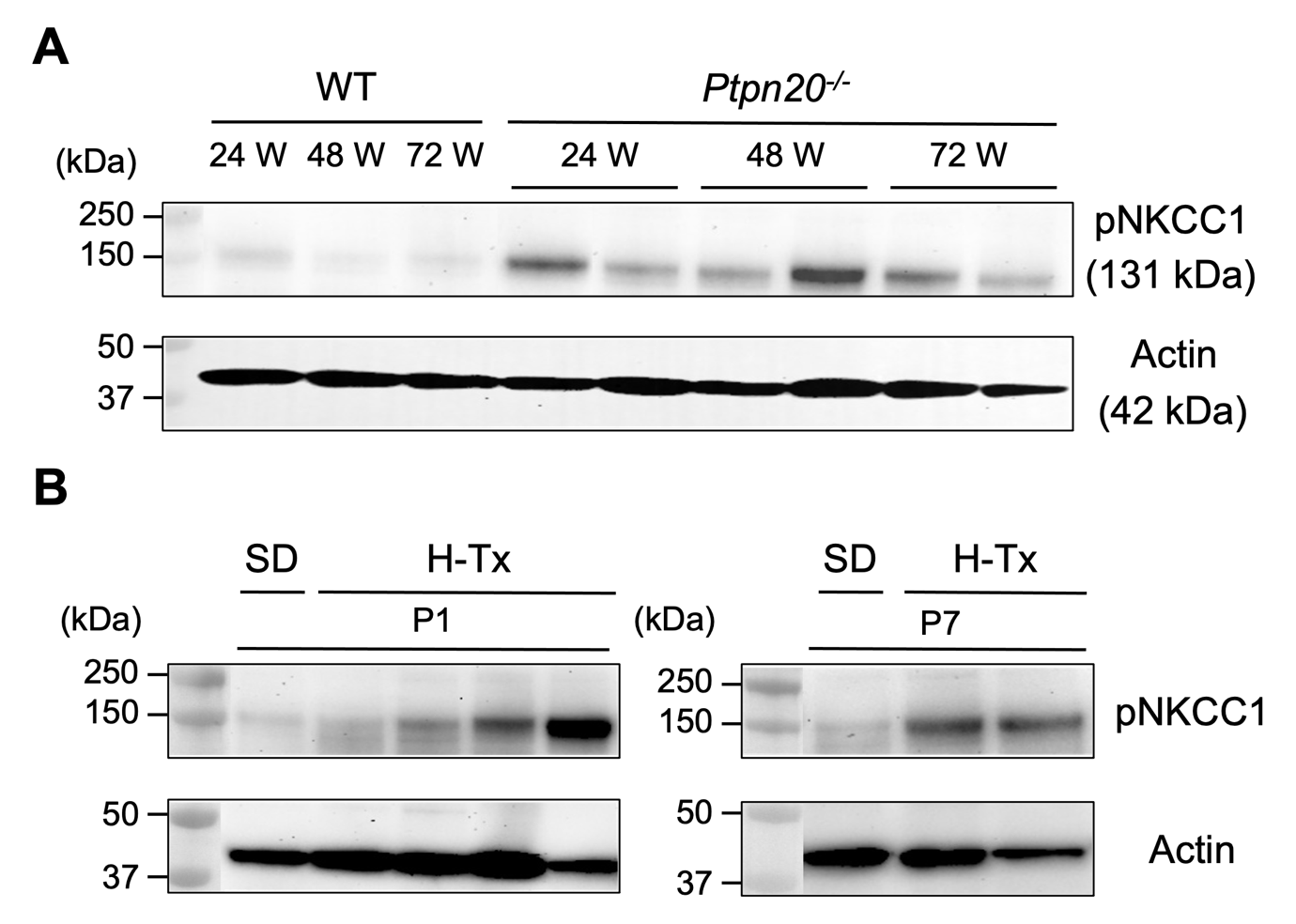 |
| --- |
| **Figure S3.** **Immunoblotting of pNKCC1 protein expression in aged *Ptpn20^-/-^* mice and H-Tx rats.**  A. Immunoblotting of pNKCC1 protein (131-kDa) expression in the choroid plexus (CP) of wild-type (WT) and *Ptpn20^-/-^* mouse at 24, 48 and 72 weeks (W). The level of pNKCC1 remained elevated for a longer period in *Ptpn20^-/-^* mice than in WT.  B. Immunoblotting of pNKCC1 protein expression in the CP of SD and H-Tx rats at P1 and P7. Expression of pNKCC1 is higher in the CP of most H-Tx rats than in SD rats. Actin (42-kDa) is used as the loading control. |
