# Supplementary material for: Ptpn20 deletion in H-Tx rats enhances phosphorylation of the NKCC1 cotransporter in the choroid plexus: an evidence of genetic risk for hydrocephalus in an experimental study
Source: Fluids Barriers CNS. 2022 Jun 3;19:39. doi: 10.1186/s12987-022-00341-z (PMC9164390; doi:10.1186/s12987-022-00341-z)
Supplement: Supplementary file 4 — Additional file 4: Table S1. Copy number variations. [file 12987_2022_341_MOESM4_ESM.docx]

**Table S1. Copy number variations**

|  |  |  | |  | CNV frequency | | |
| --- | --- | --- | --- | --- | --- | --- | --- |
| Gene name | **Full name** | **Chr.** | **CNV type** | | **H-Tx (-) (n=8)** | **H-Tx (+) (n=4)** | |
| *Hbb* | Hemoglobin subunit beta | 1 | Loss | | 8 (100%) | | 4 (100%) |
| *Pde10a* | Phosphodiesterase 10A | 1 | Loss | | 8 (100%) | | 4 (100%) |
| *Sult2a2* | Sulfotransferase family 2A, dehydroepiandrosterone (DHEA)-preferring, member 2 | 1 | Loss | | 8 (100%) | | 4 (100%) |
| *Sult2a1* | Sulfotransferase family 2A member1 | 1 | Loss | | 8 (100%) | | 4 (100%) |
| *Vom2r31* | Vomeronasal 2 receptor, 31 | 1 | Gain | | 8 (100%) | | 4 (100%) |
| *Fam111a* | FAM111 trypsin like peptidase A | 1 | Gain | | 8 (100%) | | 4 (100%) |
| *Aldh1a7* | Aldehyde dehydrogenase family 1, subfamily A7 | 1 | Gain | | 8 (100%) | | 4 (100%) |
| *RGD1309403 (Proser1)* | Proline and serine rich 1 | 2 | Loss | | 7 (87.5%) | | 3 (75%) |
| *Schip1* | Schwannomin interacting protein 1 | 2 | Loss | | 6 (75%) | | 3 (75%) |
| *Ppp3ca* | Protein phosphatase 3 catalytic subunit alpha | 2 | Loss | | 8 (100%) | | 4 (100%) |
| *Emcn* | Endomucin | 2 | Gain | | 0 (0) | | 1 (25%) |
| *Dpyd* | dihydropyrimidine dehydrogenase | 2 | Gain | | 0 (0) | | 1 (25%) |
| *Lrp1b* | LDL receptor related protein 1B | 3 | Loss | | 8 (100%) | | 4 (100%) |
| *Meis2* | Meis homeobox 2 | 3 | Gain | | 0 (0) | | 1 (25%) |
| *Cyp4a2* | Cytochrome P450, family 4, subfamily a, polypeptide 2 | 5 | Loss | | 8 (100%) | | 4 (100%) |
| *Spt1* | Salivary protein 1 | 7 | Loss | | 8 (100%) | | 4 (100%) |
| *Ncam1* | Neural cell adhesion molecule 1 | 8 | Loss | | 7 (87.5%) | | 4 (100%) |
|  |  |  | |  | **CNV frequency** | | |
| Gene name | **Full name** | **Chr.** | **CNV type** | | **H-Tx (-) (n=8)** | **H-Tx (+) (n=4)** | |
| *Trpv1* | Transient receptor potential cation channel, subfamily V, member 1 | 10 | Loss | | 5 (62.5%) | | 4 (100%) |
| *Pcp4* | Purkinje cell protein 4 | 11 | Gain | | 1 (12.5%) | | 2 (50%) |
| *Mb21d2* | Mab--21 domain containing 2 | 11 | Gain | | 7 (87.5%) | | 4 (100%) |
| *Etv5* | ETS variant transcription factor 5 | 11 | Gain | | 8 (100%) | | 4 (100%) |
| *LPP* | LIM domain containing preferred translocation partner in lipoma | 11 | Loss | | 0 (0) | | 1 (25%) |
| *Hrg* | Histidine-rich glycoprotein | 11 | Loss | | 6 (75%) | | 4 (100%) |
| *Xpr1* | Xenotropic and polytropic retrovirus receptor 1 | 13 | Loss | | 8 (100%) | | 4 (100%) |
| *Vom2r69* | Vomeronasal 2 receptor, 69 | 14 | Loss | | 8 (100%) | | 4 (100%) |
| *Lphn3* | Latrophilin 3 | 14 | Gain | | 0 (0) | | 1 (25%) |
| *Mcpt3* | Mast cell peptidase 3 | 15 | Loss | | 8 (100%) | | 4 (100%) |
| *Ptpn20* | Protein tyrosine phosphatase, non-receptor type 20B (Ptpn20b) | 16 | Loss | | 2 (25%) | | 4 (100%) |
| *Dlgap2* | DLG associated protein 2 | 16 | Gain | | 7 (87.5%) | | 4 (100%) |
| *Cyp4v3* | Cytochrome P450, family 4, subfamily v, polypeptide 3 | 16 | Gain | | 8 (100%) | | 4 (100%) |
| *Slc10a2* | Solute carrier family 10-member 2 | 16 | Gain | | 8 (100%) | | 4 (100%) |
| *Stard3nl* | STARD3 N-terminal like | 16 | Gain | | 8 (100%) | | 4 (100%) |
| *Palldl1* | palladin-like 1 | 16 | Loss | | 8 (100%) | | 4 (100%) |
| *Adarb2* | Adenosine deaminase, RNA-specific, B2 | 17 | Loss | | 8 (100%) | | 4 (100%) |
|  |  |  | |  | **CNV frequency** | | |
| Gene name | **Full name** | **Chr.** | **CNV type** | | **H-Tx (-) (n=8)** | **H-Tx (+) (n=4)** | |
| *Akr1c12l1* | Aldo-keto reductase family 1, member C12-like 1 | 17 | Loss | | 8 (100%) | | 4 (100%) |
| *Eci2* | EnoylCoA delta isomerase 2 | 17 | Loss | | 7 (87.5%) | | 4 (100%) |
| *RGD1308114 (Sugct)* | Sugct succinyl CoA: Glutarate-CoA transferase | 17 | Loss | | 0 (0) | | 3 (75%) |
| *Amph* | Amphiphysin | 17 | Gain | | 8 (100%) | | 4 (100%) |
| *Gpr158* | G protein-coupled receptor 158 | 17 | Loss | | 8 (100%) | | 2 (50%) |
| *Commd10* | COMM domain containing 10 | 18 | Loss | | 8 (100%) | | 4 (100%) |
| *Nedd4l* | NEDD4 like E3 ubiquitin protein ligase | 18 | Loss | | 6 (75%) | | 2 (50%) |
| *Spire2* | Spire-type actin nucleation factor 2 | 19 | Gain | | 0 (0) | | 1 (25%) |
| *Snx3* | Sorting nexin 3 | 20 | Loss | | 8 (100%) | | 4 (100%) |
| *RT1-Db1* | RT1 class II, locus Db1 | 20 | Loss | | 7 (87.5%) | | 4 (100%) |
| *RT1-Bb* | RT1 class II, locus Bb | 20 | Loss | | 8 (100%) | | 4 (100%) |
| *Herc4* | HECT and RLD domain containing E3 ubiquitin protein ligase 4 | 20 | Gain | | 0 (0) | | 1 (25%) |
| *Pkib* | cAMP-dependent protein kinase inhibitor beta | 20 | Gain | | 0 (0) | | 1 (25%) |

**Table S1.** Copy number Variations (CNVs) between eight H-Tx (-) rats, and four H-Tx (+) rats, gene name, chromosome, copy number variation type are shown for each gene. The copy number variations ratio is shown in brackets. Candidate genes are marked with a gray shading.
