# Supplementary material for: Ptpn20 deletion in H-Tx rats enhances phosphorylation of the NKCC1 cotransporter in the choroid plexus: an evidence of genetic risk for hydrocephalus in an experimental study
Source: Fluids Barriers CNS. 2022 Jun 3;19:39. doi: 10.1186/s12987-022-00341-z (PMC9164390; doi:10.1186/s12987-022-00341-z)
Supplement: Supplementary file 5 — Additional file 5: Table S2. Assay of real-time PCR. [file 12987_2022_341_MOESM5_ESM.docx]

**Table S2. Assay of Real-time PCR**

| **Animals** | **Target** | **Assay ID (Thermo Fisher Scientific)** |
| --- | --- | --- |
| Rat | *Ptpn20*  Actin | Ptpn20b, Rn01756221_m1  Actb, Rn00667869_m1 |
| Mouse | *Ptpn20*  AQP1  NKCC1  Na^+^, K^+^-ATPase  Actin | Ptpn20, Mm00477234_m1  AQP1, Mm00431834_m1  NKCC1, Mm01265951_m1  ATP1a1, Mm00523255_m1  Actb, Mm00607939_S1 |
