# Supplementary material for: Ptpn20 deletion in H-Tx rats enhances phosphorylation of the NKCC1 cotransporter in the choroid plexus: an evidence of genetic risk for hydrocephalus in an experimental study
Source: Fluids Barriers CNS. 2022 Jun 3;19:39. doi: 10.1186/s12987-022-00341-z (PMC9164390; doi:10.1186/s12987-022-00341-z)
Supplement: Supplementary file 6 — Additional file 6: Table S3. Primary antibodies forimmunoblot investigations. [file 12987_2022_341_MOESM6_ESM.docx]

**Table S3. Primary antibodies for immunoblot investigations**

| **Animal** | **Protein** | **Target** | **Molecular weight** | **Primary antibodies** |
| --- | --- | --- | --- | --- |
| Rat | 10 µg | *Ptpn20* | 50 kDa | Rabbit Ptpn20 antibody 48kDa (orb317724, Biorbyt, Cambridge, UK, 1:500) |
|  |  | Actin | 42 kDa | Rabbit Anti-Actin 42kDa (A2066 SIGMA, Saint Louis, Missouri 63103 USA, 1:200) |
| Mouse | 10 µg | *Ptpn20* | 50 kDa | Rabbit Ptpn20 antibody 48kDa (orb317724, Biorbyt, Cambridge, UK, 1:500) |
|  | 15 µg | pNKCC1 | 131 kDa | Rabbit phospho-NKCC1 antibody (Thr212/Thr217) 131kDa (ABS1004, Sigma-Aldrich, Darmstadt, Germany, 1: 500) |
|  |  | Actin | 42kDa | Rabbit Anti-Actin 42kDa (A2066 SIGMA, Saint Louis, Missouri 63103 USA, 1:200) |
