# Supplementary material for: Ptpn20 deletion in H-Tx rats enhances phosphorylation of the NKCC1 cotransporter in the choroid plexus: an evidence of genetic risk for hydrocephalus in an experimental study
Source: Fluids Barriers CNS. 2022 Jun 3;19:39. doi: 10.1186/s12987-022-00341-z (PMC9164390; doi:10.1186/s12987-022-00341-z)
Supplement: Supplementary file 7 — Additional file 7: Table S4. Reagents forimmunofluorescence investigations. [file 12987_2022_341_MOESM7_ESM.docx]

**Table S4. Reagents for immunofluorescence investigations**

| **Animal** | **Method** | **Target** | **Reagents** | |
| --- | --- | --- | --- | --- |
|  |  |  | **Primary antibody** | **Secondary antibody** |
| Rat | Paraffin | *Ptpn20* | Rabbit PTPN20B antibody (CSB-PA065165, CusAb, 1:250) | Alexa Fluor® 488 donkey anti-rabbit IgG (A21206, Molecular Probes®, Invitrogen, 1:250) |
| Mouse | Vibratome | pNKCC1 | Rabbit pNKCC1 antibody (CSB-PA065165, CusAb, 1:25) | Alpaca anti-Rabbit IgG AlexaFluor488 (SA510322: Thermo, 1:100) |
|  |  | NKCC1 | Goat NKCC1 antibody (C-14) sc-21547 (Santa Cruz Biotechnology, 1:25) | Donkey anti-Goat IgG (H+L) Alexa Fluor^TM^ Plus 594 (A32758, Invitrogen, 1:50) |
|  |  | AQP1 | Mouse AQP1 (B-11) sc-25287 (Santa Cruz Biotechnology, 1:25) | Donkey anti-Mouse IgG (H+L) Alexa Fluor^TM^ Plus 488 (A32766, Invitrogen, 1:25) |
|  |  | Na^+^/K^+^-ATPase | Goat Na^+^/K^+^-ATPase a1 (C-20) sc-16043 (Santa Cruz Biotechnology, 1:25) | Donkey anti-Goat IgG (H+L) Alexa Fluor^TM^ Plus 594 (A32758, Invitrogen, 1:50) |
|  |  | E-Cadherin | Mouse Anti-E-Cadherin (Becton, Dickinson and Company, 1:200) | Donkey anti-Mouse IgG (H+L) Alexa Fluor^TM^ Plus 594 (A32744, Invitrogen, 1:50) |
|  | Cryosection | *Ptpn20* | Rabbit PTPN20B antibody (CSB-PA065165, CusAb, 1:250) | Alexa Fluor® 488 donkey anti-rabbit IgG (A21206, Molecular Probes®, Invitrogen,1:250) |
|  |  | F-Actin | Actin-stain 555 Fluorescent Phalloidin (Cat. # PHDH1, Cytoskeleton. Inc., 1:50) | |
